# Efficacy of Government-Sponsored Community Health Programs for Older Adults: A Systematic Review of Published Evaluation Studies

**DOI:** 10.3389/phrs.2022.1604473

**Published:** 2022-09-23

**Authors:** Arun Chandrashekhar, Harshad P. Thakur

**Affiliations:** Tata Institute of Social Sciences, Deonar, India

**Keywords:** older adults, global health, program evaluation, aging, community health

## Abstract

**Objective:** Population aging is an ongoing challenge for global health policy and is expected to have an increasing impact on developing economies in years to come. A variety of community health programs have been developed to deliver health services to older adults, and evaluating these programs is crucial to improving service delivery and avoiding barriers to implementation. This systematic review examines published evaluation research relating to public and community health programs aimed at older adults throughout the world.

**Methods:** A literature search using standardized criteria yielded 58 published articles evaluating 46 specific programs in 14 countries.

**Results:** Service models involving sponsorship of comprehensive facilities providing centralized access to multiple types of health services were generally evaluated the most positively, with care coordination programs appearing to have generally more modest success, and educational programs having limited effectiveness. Lack of sufficient funding was a commonly-cited barrier to successful program implementations.

**Conclusion:** It is important to include program evaluation as a component of future community and public health interventions aimed at aging populations to better understand how to improve these programs.

## Introduction

The global population is aging rapidly, with the number of older adults worldwide projected to more than double in the coming decades to more than 1.5 billion [1]. The aging population is increasing in all 195 countries in the world, with the fastest rate of aging shifting away from Europe and North America and toward China and India [2]. These trends have important implications for public health, as the burden of diseases related to older age, ranging from cardiovascular disease [3], to cancer [4], to diabetes [5], to dementia [6], are expected to continue to increase. In addition to the impact of these public health challenges on population well-being, they are expected to cause substantial increases in public and private expenditures on health care [1]. The global burden of these increases in health care demand are likely to have the most severe impact on low and middle income countries with less developed health care infrastructures [7]. However, countries at all levels of development are fundamentally impacted, making it essential to address solutions at a global level. To improve population health and decrease the costs of medical care in late life, governments around the world have undertaken programs aimed at addressing the specific medical needs of older adults. The purpose of this study is to systematically review the empirical literature at the global level evaluating government-funded community health programs addressing the health of older adults.

### The Demographic Transition Model and Global Population Aging

Population change is a function of the rates of birth and mortality [8]. Advances in medical care and public health over the course of the twentieth and twenty-first centuries resulted in globally declining mortality rates, and consequently to exponential increases in the world population [9]. It is estimated that it took until the year 1800 for the global population to reach 1 billion people; the global population subsequently more than doubled in the next 150 years [9] and reached 7 billion by 2011 [10]. However, this growth has not been uniform throughout the world. Regions seeing the earliest decreases in mortality rates also began to see subsequent decreases in birth rates, such that net natural population growth has halted or turned to decline in much of the global North [9]. In the global South, where changes in the mortality rate accrued later due to long-term structural economic and material inequalities, changes in birth rates have only started to begin to change [11]. As a result, it is estimated that the global population will reach a maximum of approximately 11 billion by the year 2100, before declining as the global birth rate falls below the global death rate [12].

In the interim, these population dynamics dictate that the age composition of the population is unequal between regions globally and remains in a state of long-term change throughout the world. These patterns have been identified for many decades and are collectively known as the demographic transition model [8]. In particular, the combination of declining birth rate and increasing life span in the global North has led to dramatic population aging, with the number of older adults exceeding the number of young people in some areas [9]. Meanwhile, a combination of falling mortality, rising life expectancy, and continued high birth rates in the global South have resulted in a sustained period of high population growth in these regions, with very young median ages [11]. The result has been countervailing population pressures in these two global regions. The large number of older adults relative to younger and middle adults in the global North has created a high dependency ratio, with relatively few working-age adults to support the population of pensioners, placing significant strain on social service and health care budgets [9]. In the global South, the large population of younger adults has created strains on educational and economic resources [13]. A result has been substantial population migration from the global South to the global North [12].

As demographic trends continue to evolve, new sources of strain can be anticipated. As inequalities in access to medical and educational resources in the global South have begun to be levelled, so too has the birth rate in these regions begun to decline, in a reflection of trends seen in previous generations in the global North [9, 12]. Thus, it can be anticipated that population aging will follow an analogous trajectory in these regions [14], with similar implications for increasing burdens of health care expenditures. Countries faced with the expectancy of these transitions have an interest in developing solutions to reduce the health care burdens associated with an aging population. For example, India began implementing the National Programme for the Health Care of the Elderly (NPHCE) during the last decade to provide integrated medical services to older adults through funding facilities improvements at the regional level [15]. The aim of this study is to systematically review all available evaluation studies of government-funded community health programs for older adults implemented. Based on the demographic transition model, it is anticipated that adoption of such programs in the global South has increased in recent years.

## Methods

A systematic review was conducted according to the Preferred Reporting Items for Systematic Review and Meta-Analyses (PRISMA) standard, which were initially established in 2009 and has been endorsed by a large body of scientific journals and editorial consortia [16]. To identify qualifying publications, a literature search was conducted using the following inclusion and exclusion criteria. Articles were included in the review if they 1) reported on the evaluation of one or more community health programs 2) carried out by or with the support of a governmental body at the local, regional, or national level and 3) specifically serving an older adult population, and that 4) were published in English 5) between 2000 and December 31, 2020. Reports on single-institution clinical studies were excluded. Excluded item types included 1) theses or dissertations, 2) conference abstracts and proceedings, 3) theoretical papers, 4) comments or letters to the editor, and 5) previous reviews. To identify qualifying articles, a literature search was conducted in Google Scholar, PubMed, and Science Direct, using a combination of search criteria specifying the scope each element of the review (see Table 1). These three databases were used because they collectively index of more than 30,000 journals in the medical and social sciences, providing comprehensive coverage of the relevant published literature.

**TABLE 1:** Literature search criteria (Global, 2000–2020).

Criterion	Search terms
Older adult focused	Elderly; older adult; geriatric
Policies and programs	Policy; program
Community health design	Community model; community-based model; community health
Evaluation	Evaluation; outcomes; efficacy
Implemented broadly in an official capacity	Government; national; regional; state; provincial; municipal

## Results

**FIGURE 1:**
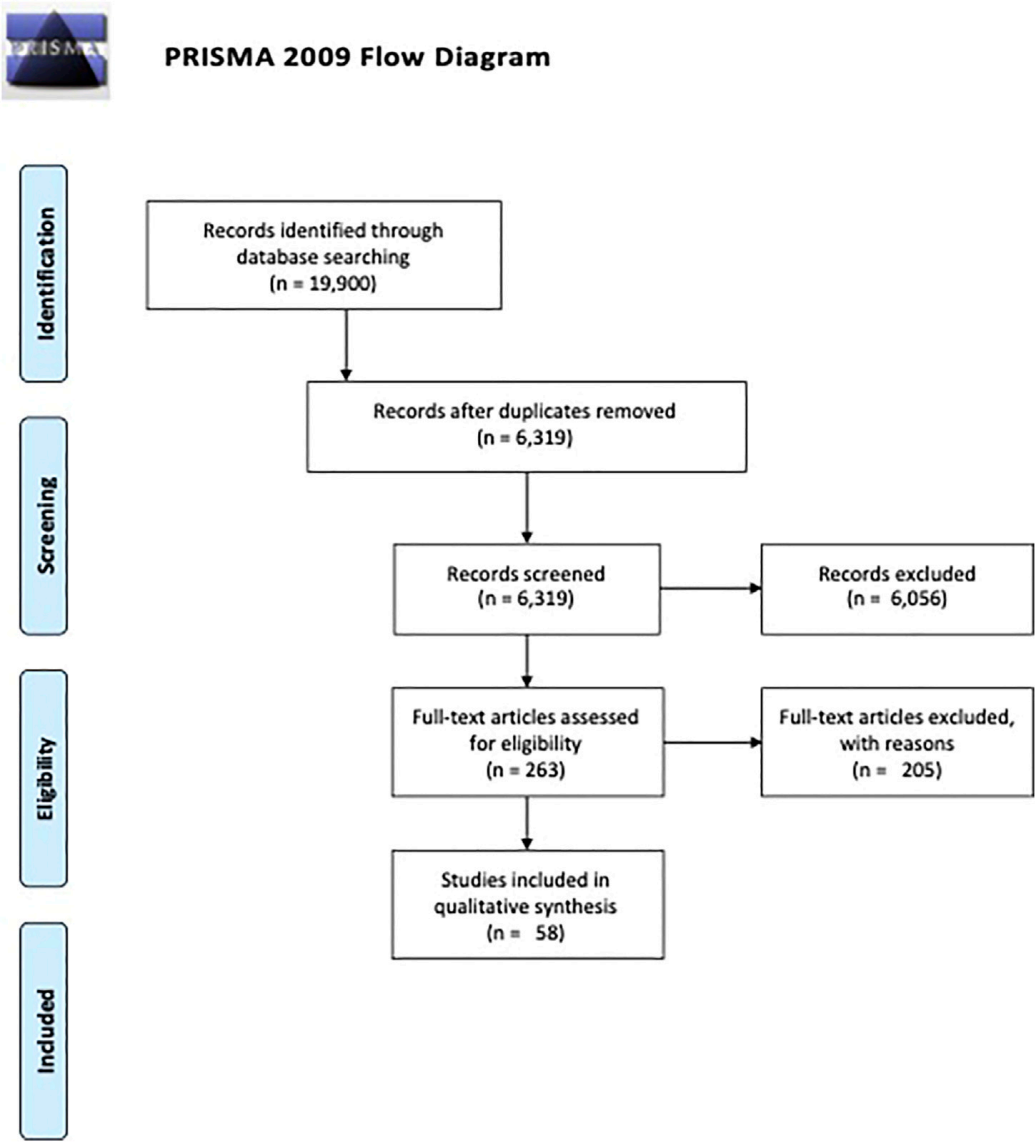
Preferred reporting items for systematic review and meta-analyses flow diagram (Global, 2000–2020).

Figure 1 presents a flow diagram of the literature search and screening process. From an initial pool of 19,900 search results, a final sample of 58 articles meeting all of the review criteria were identified and included in this review. Details and key findings of these studies are summarized in Table 2. In terms of geography, 22 studies evaluated programs located in the United States (37.9%), 9 in Japan (15.5%), 6 were in Canada (10.3%), 3 each in Taiwan and Denmark (5.2% each), 2 each in Brazil, China, Indonesia, and Netherlands (3.5% each), and 1 each in Australia, Bangladesh, Mexico, Sweden, and United Kingdom (1.7% each). In terms of program scope, 23 were conducted at the community level (40.4%), 17 at the national level (29.3%), 11 at the State or Provincial level (19.3%), and 7 at the regional level (12.3%).

**TABLE 2:** Summary of program evaluations matching search criteria (Global, 2000–2020).

Authors	Program location	Program scope	Evaluation methodology	Program description	Key findings
Ackerman et al. [17]	Washington State, United States	Regional	Case-control	Program implemented by private health insurance company administering benefits to Medicare (public health insurance for older adults) to provide voluntary exercise classes	Program participants had lower health care costs after 2 years in comparison to a matched group of non-participants. Savings were greatest among the most frequent participants
Agarwal et al. [18]	Ontario, Canada	Provincial	RCT (planned)	Planned pilot study providing education on preventive and emergency health to older adults living in government-subsidized housing	Evaluation planned but not implemented. Planned endpoints include reduction in emergency service use and change in health behaviors
Allen et al. [19]	Ohio, United States	Regional	Descriptive	Innovation Center Model for integrating Federally-funded and privately-funded aging care programs under a single planning structure within regional health care systems	A summary of program outcomes is provided, including patient education and improved clinical outcomes for older adults and their caregivers
An [20]	United States	National	Nutrition survey	Federal program funding delivery of prepared meals to isolated and homebound older adults (Meals on Wheels)	Older adults had improved nutritional intake from home-delivered meals compared with other sources
Bartels et al. [21]	United States	National	Descriptive	Several programs funded under the Affordable Care Act (ACA) to provide mental health care services for older adults	The ACA aims to improve geriatric mental health care through a combination of direct funding and mandated quality improvement projects for providers. Some programs are not fully implemented nationally due to lack of funding
Beeber [22]	Northeastern United States	Regional	Patient interviews	The Program of All-Inclusive Care for the Elderly (PACE) provides federal funding for assessment, service coordination, and home-based care for older adults eligible for nursing home care	The PACE program effectively meets the needs of some older adults, but enrolment is limited by a lack of public knowledge of program availability. Low adoption by patients may contribute to limited implementation by regional health systems
Belza et al. [23]	United States	Multi-Regional	Descriptive	Pilot program designating and funding regional Health Aging Research Network (HAN) centers to evaluate and promote community-based public health initiatives	The HAN model increased collaboration within communities and was effective at making training and implementation materials for healthy aging program available in the areas in which it was piloted
Bernabei [24]	Italy	Multi-regional	Descriptive	The Progetto Obiettivo Salute dell’Anziano (POSA) established the goal of creating integrated networks of social and health services for older adults to be administered in the context of regional geriatric centers	Implementation of POSA has been limited to only some regions, due to funding constraints. Three partial evaluations support the efficacy of the POSA components
Chambers et al. [25]	Ontario, Canada	Community	Patient survey	Pilot Cardiovascular Health Awareness Program (CHAP) education and screening program administered in pharmacies in two communities	Patient satisfaction was high, and program participation resulted in increased willingness of patients to discuss cardiovascular health issues with their primary care physicians
Cheadle et al. [26]	Seattle, Washington, United States	Community	Expert interviews and program records	The Southeast Seattle Senior Physical Activity Network (SESPAN), a community organizing program to increase opportunities for older adults to participate in physical activity by networking between local nonprofit organizations and businesses	The SESPAN program contributed to the implementation of 21 exercise programs for older adults as well as leading to improvements in community walkability
Chiang and Hsu [27]	Taiwan	National	Patient and expert interviews	The Community Care Centres Plan (CCCP) establishes local community centers for older adults as well as home visitation for delivery of health promotion resources and social support	Older adults who participated in the CCCP reported improved health and social capital
De Vlaming, et al. [28]	Netherlands	Community	Planned community-level case-control comparison	Pilot “Healthy Aging” program providing public information, education, and health activities for older adults with an aim of reducing loneliness	Evaluation planned but not implemented. Planned evaluations include examination of program records and a case-control survey between pilot community and control community
Duke [29]	North Carolina, United States	Community	Pre/post measurement of hospital resource use	Pilot program providing case management integrating community social services and clinical care for older adults	Emergency and hospital use and costs were reduced in the target population after program implementation
Forti and Koerber [30]	South Carolina, United States	Community	Records review and patient surveys	Pilot program providing case management and coordination of social services and clinical care for older adults, focused on serving a rural African American population	More than half of participants were enrolled in social service programs. Participants reported better access to food and health care after enrolling
Goeree et al. [31]	Ontario, Canada	Multi-community	Randomized controlled trial	Cardiovascular Health Awareness Program (CHAP) education and screening program administered in pharmacies in 39 communities	Hospitalization costs for cardiovascular disease were reduced among the target population in communities participating in the trial compared to controls
Gyurmey and Kwiatkowski [32]	Rhode Island, United States	State	Program records	The Program of All-Inclusive Care for the Elderly organization of Rhode Island (PACE-RI) administers the federal PACE program (see [22])	PACE-RI participants live at home rather than in a nursing home for an average of 4.3 years, and have 11% fewer emergency visits compared with similar non-enrollees
Honigh-de Vlaming et al. [33]	Netherlands	Community	Population surveys	Pilot “Healthy Aging” program providing public information, education, and health activities for older adults with an aim of reducing loneliness (see also de Vlaming et al. [28])	Awareness of loneliness issues was significantly better in the intervention community, but there were no significant differences in social support
Imamura [34]	Ohio, United States	Community	Participant surveys	Pilot community nursing program to provide health information and facilitate social support relationships among older adults	Participants were satisfied with the program and continued to engage in physical activities after the program ended
Ippoliti et al. [35]	Italy	Regional	Economic analysis	The Community Nurse Supporting Elderly in a changing Society (CONSENSO) program is funded by the European Union in an effort to provide community nursing services to older adults living in rural mountain areas	The CONSENCO program can pay for itself in reduced medical costs by preventing 1,657 hip fractures in the target geographic region over the course of 3 years
Kadar et al. [36]	Gowa District, Indonesia	Regional	Patient survey and provider interviews	The Indonesian government provides social support and health services aimed at older adults through local community health centers	Many services are not available outside of urban areas due to funding limitations
Kane et al. [37]	Minnesota, United States	State	Case-control medical records review	The Minnesota Senior Health Option (MSHO) provides preventative and community medicine services to economically-disadvantaged older adults	MSHO participants had a lower rate of emergency department visits and preventable hospitalizations compared with matched controls
Kronborg et al. [38]	Denmark	National	Randomized controlled cost effectiveness analysis	Home health visits for screening and preventive medicine by community health providers are funded by the Danish government and administered by municipalities. Differences in program design between localities include the level of integration with patients’ general practitioners	Participation in the preventive home visit program resulted in more mean active life years for older adults, but did not impact medical costs
Larsen et al. [39]	Randers, Denmark	Municipal	Program records	Three alternative community-based pilot fall prevention programs were tested in a randomized controlled trial, including a home visit focused on residential safety and diet; provision of vitamin supplements; or both	There were no differences in the acceptance rates for each of the three program designs
Lawson et al. [40]	Nova Scotia, Canada	Provincial	Provider and patient interviews	Feasibility study of the implementation of a care coordination and screening program for frail older adults	Use of the screening program increased older adults’ visit times with primary care providers. Users were satisfied with the program
Lawson et al. [41]	Nova Scotia, Canada	Provincial	Descriptive	Planned implementation of a care coordination and screening program for frail older adults	Interviews with patients and providers are planned to evaluate program success
Li et al. [42]	Yuexiu District, Guangzhou, China	Municipal	Randomized controlled trial	Pilot community health program aimed at older adults with hypertension and including educational, and disease management support components, with support mediated by WeChat social media	Program participants achieved better control of blood pressure compared with matched controls
Liang et al. [43]	Taiwan	National	Medical records	Community Care Station (CCS) provide recreational and community health services to older adults; this study evaluates structured exercise programs offered in some CCS locations	Participants in structured exercise programs improved their physical fitness and maintained these improvements over a two-year follow-up period
Lindqvist et al. [44]	Motala, Sweden	Municipal	Program records and community health statistics	Public health program to improve fall-related public safety infrastructure, provide public safety communication, and assist older adults with fall safety home improvements	The community rate of moderate fall-related injury decreased after program implementation, but the rate of severe and fatal falls did not change; risk was reduced among younger-old but not older-old residents
Liu et al. [45]	Changsha, China	Municipal	Community interviews	Feasibility study for proposed implementation of an evidence-based fitness program for older adults	Potential barriers to implementation include lack of access to resources and lack of awareness of health issues affecting older adults
Lowe and Coffey [46]	Northern Territory, Australia	State	Program records	Community health programs for older adults include the Commonwealth Home Support Program (CHSP), which provides home health services for patients not requiring coordination of care, and the Home Care Packages (HCP) program serving patients with more complex care management needs	Population aging is expected to lead to increased service use. Disparities between aboriginal and non-aboriginal older adults is particularly acute for nutrition services
Luzinski et al. [47]	Fort Collins, Colorado, United States	Municipal	Economic analysis	Community case management program for high-risk older adults designed to improve preventive care and coordination of care	Hospital costs were reduced due to reduction of unreimbursed emergency care
Martinez-Maldonado et al. [48]	Valle del Mezquital, Hidalgo, Mexico	Municipal	Provider interviews	Community health program in which older adults were trained as health promoters to assist other older adults with access to medical and social services	Success was dependent on promoters’ access to social capital and position within their communities
Matsudo et al. [49]	Sao Paulo State, Brazil	State	Program records, participant surveys	A multi-level public health campaign to increase physical activity of which older adults are one of the three primary groups targeted by specific interventions, including health communication and organization of events	Older women increased their physical activity after program participation
Matsudo et al. [50]	Sao Paulo State, Brazil	State	Public survey	A multi-level public health campaign to increase physical activity of which older adults are one of the three primary groups targeted by specific interventions, including health communication and organization of events	Public knowledge and retention of public health messaging was high after a four-year program period
McCarrell [51]	United States	National	Descriptive	PACE program (see [22])	Pharmacy services are not integrated in PACE programs in many areas, but it is suggested that older adults would benefit from more participation by pharmacists in care coordination
Mukamel et al. [52]	Monroe County, New York, United States	Municipal	Cost-benefit analysis	Public health program providing pneumococcal vaccination for older adults	Vaccination of older adults outside of clinic settings can be effective for increasing overall vaccination rates, but is associated with higher costs relative to vaccination in clinic settings
Murashima and Asahara [53]	Japan	Municipal	Case-control comparison at the municipal level based on medical admissions records	Home health care programs administered at the local level; in some places, this includes use of a combination of community nursing and family resources to provide home-based care for older adults called around the clock care (ACC)	A municipality implementing ACC was successful in lowering the rate of institutionalization of older adults in comparison with a control municipality with no ACC program
Nakamura et al. [54]	Japan	National	Program records and provider surveys	A public-private partnership program designed to encourage convenience store employees to assist in screening for health problems in older adult customers	Regional differences in plan implementation are reflected in differences in reported assistance provided to older adults by employees. Program participation increased the rate at which older adults received assistance
Nakanishi et al. [55]	Japan	National	Medical records	The Orange Plan was adopted by the Japanese government to provide coordination of care services for older adults with dementia, with the goal of increasing the rate of home-based care	The rate of dementia deaths occurring in institutions rather than at home continued to increase at the same rate after program implementation
Olivares-Tirado et al. [56]	Japan	National	Medical records	The Japanese system of Long Term Care Insurance (LTCI) includes coordination of care and other case management and home health services for disabled older adults. This study examines transition services aimed at improving physical functioning in new disability patients	LTCI services did not results in substantial improvements in functional status, suggesting changes should be made in the program
Otaka et al. [57]	Tatebayashi City, Japan	Municipal	Medical records	Recreation and health promotion activities provided by local senior citizen centers (“community salons”). A fall prevention program is specifically evaluated	Participation in the fall prevention program was associated with a significant reduction in fall risk
Oyama et al. [58]	Joboji, Japan	Municipal	Mortality statistics	Community-based suicide prevention program for older adults implementing depression screening and psychiatric referrals	The suicide rate among older adults was reduced by approximately 25% in the implementation area compared with a matched control region with no program implementation
Pacala et al. [59]	United States	National	Patient survey	PACE program (see [22])	Most patients were satisfied with the quality of care received, and most sites were evaluated as providing better care than non-PACE alternatives
Ploeg et al. [60]	Ontario, Canada	Municipal		The Homelessness Intervention Program (HIP) provides coordination of care and case management services to homeless older adults in parts of Ontario	The program was effective at improving continuity of care for participants, but more systemic changes are called for to reduce homelessness
Rahmawati and Bajorek [61]	Indonesia	National	Provider interviews	The Integrated Health Service Post for the Elderly (IHSP-Elderly) provides community health worker services for screening and case management of older adults. This study evaluates a hypertension management program sponsored under the IHSP program	The program was successful at improving older adults ability to manage their hypertension, but effectiveness was limited by geographic disparities in access to IHSP services
Rana et al. [62]	Chandpur District, Bangladesh	Municipal	Program records; participant surveys	Pilot community-based public health education program regarding nutrition and chronic disease management tested in eight rural villages in Bangladesh	Program participants experienced significant improvements in health knowledge, condition management, and quality of life
Sakayori [63]	Japan	National	Medical records	The Long-Term Care Prevention Project provides community health support with the aim of preventing the need for institutional care in older adults. The Exercises for Healthy Oral Function program is part of this program, and provides screening and therapy to treat early signs of difficulty with speech and eating	Patients enrolled in the program improved oral function after 1 year
Segelman et al. [64]	United States	National	Medical records	Comparison of older adults receiving services under two different federal programs, PACE (see [22]), which provides a high level of service integration, and 1915c waiver program, which provides home-based services with less coordination of care	Older adults enrolled in PACE had better physical and cognitive impairment outcomes
Shinaki et al. [65]	Kusatu, Japan	Municipal	Medical records	Pilot community health frailty prevention program involving annual screening and education for older adults	Program participants experienced functional improvements, and use of residential care services increased more slowly in the service area in comparison to the rest of the country
Smith et al. [66]	United States	Multi-State	Program records	The State Falls Prevention Program (SFPP) was a federally-funded and state-administered program to introduce community-based physical activity programs for older adults	Four programs were successfully initiated. Obtaining alternate funding is a barrier to program sustainability
Tomioka et al. [67]	Kauai County, Hawaii, United States	State	Medical records	State-sponsored health promotion outreach program for older adults promoting fitness training (EnhanceFitness)	Program participants improved physical fitness outcomes and had lower hospitalization costs
Vass et al. [68]	Denmark	Municipal	Medical records	Pilot community health program providing education and screening for older adults	Program increased referrals to home care programs and resulted in improved functional ability in participants; mortality and nursing home use were not affected
Vouri et al. [69]	United States	National	Patient survey	PACE program (see [22])	New enrollees had significantly improved mood and reduced symptoms of depression after 9 months in the program
Wang et al. [70]	A-Lein township, Taiwan	Municipal	Medical records	Influenza vaccination outreach program for older adults	Vaccination decreased hospitalization costs and mortality risk for program participants
Wieland et al. [71]	United States	National	Medical records	PACE program (see [22])	Program participants had lower mortality risk than older adults lacking integrated care programs, and this effect was greatest for patients in the highest risk categories
Wildman et al. [72]	Durham, United Kingdom	Regional	Participant and provider interviews	Come Eat Together (CET) program providing older adults with opportunities for nutrition and social interaction	Program success depends on perception that the program is tailored to the needs of the community and that there has been community involvement in program design
Wright et al. [73]	United States	National	Participant survey	Meals on Wheels program	Participant nutrition and mental health improved after program enrollment
Yasunaga et al. [74]	Japan	National		Research on Productivity through Intergenerational Sympathy (REPRINTS) program aimed at improving older adults’ health and well-being outcomes through establishing volunteering relationships with primary schools	Program participants had better self-rated health than controls

A diverse range of evaluation methodologies were employed, with some studies implementing multiple evaluation techniques. The most commonly used evaluation tool was review of participant medical records to examine whether or not program participants had lower demand for health services and/or better health outcomes in comparison to others; this methodology was applied in 13 studies (22.4%). Other common methodologies included participant surveys (11 studies, 19.0%), program record reviews (10 studies, 17.2%), provider interviews (8 studies, 13.8%), and participant interviews (6 studies, 10.3%). Other studies employed prospective designs to evaluate pilot programs using randomized controlled trials (4 studies, 6.9%) or a case-control comparison with matched communities (4 studies, 6.9%). Six studies were primarily descriptive and enumerated program successes and failures without necessarily employing a formal evaluation methodology (10.3%). Other studies used economic analysis (4 studies, 6.9%), public opinion surveys including non-participants as well as program participants (2 studies, 3.5%), and examination of community-level health outcomes (2 studies, 3.5%).

Some programs were evaluated by multiple studies. In particular, 7 articles [17–23] addressed the PACE program, which is a longstanding federally sponsored health program in the US that encourages the establishment of coordinated care services for older adults. Several other programs were evaluated by two studies each, including the US Meals on Wheels program [24, 25], the Community Care Centres Plan (CCCP) in Taiwan [26, 27], the Cardiovascular Health Awareness Program (CHAP) in Ontario [28, 29], the Agita Sao Paulo Program in Brazil [30, 31], the Frailty Portal program in Nova Scotia [32, 33], and the Healthy Aging program in the Netherlands [34, 35]. In the last four of these cases, this represented an early descriptive evaluation of the program, followed by a second more formal evaluation.

The approach and range of services offered by the evaluated programs was very diverse. The most comprehensive programs were designed to establish centers providing comprehensive health care for older adults, usually with the goals of reducing the need for emergency medical intervention and allowing older adults to receive care in their own homes for as long as possible, rather than requiring residential nursing home care. There was a total of 9 studies examining these programs, including 8 addressing the PACE program in the US and 1 addressing the POSA program in Italy. Other programs with similar aims were designed to coordinate care between multiple existing health care and social service providers, rather than combining these services under the authority of a single center. There were also 9 studies of programs of this type, including 3 in the US, 3 in Canada, 2 in Japan, and 1 in Australia. A program based in Mexico was designed to facilitate coordination of care through the use of volunteer peer health navigators [36]. A conceptually related type of program aimed to create senior activity centers providing a combination of recreational, social, and health care services for older adults. Programs of this type were evaluated by studies located in Taiwan [26, 27] and Indonesia [37]. The distribution of countries represented here was derived from the literature search as described above, and represents the programs with qualifying published evaluations.

Other community health programs with more limited scope included health visitation and screening programs [38–43], mental health service programs [44], vaccination programs [45, 46], meal programs [24, 25, 47], and prevention of specific health problems including falls [48–51], hypertension [37, 52], and suicide [53]. Finally, less comprehensive and time-limited community health programs including education and fitness classes accounted for 18 of the programs evaluated (31.0%).

## Discussion

This review highlights two important elements of community health intervention for older adults: the extent of program implementation throughout the globe, and the application of evaluation frameworks to these programs. Regarding the first issue, it is clear that governmental authorities throughout the world have acknowledged the importance of implementing programs aimed at serving the older adult population as a key element of addressing the challenges of population aging. The programs covered by the evaluations reviewed here represent five continents, including both developed and developing economies. Many of these programs are clustered in countries where population aging has been a longstanding concern, including Japan, Italy, Canada, and the US. Countries with younger populations tend to have less developed economies [1], making it difficult to determine whether the under-representation of these countries in the evaluations covered in this review may be due to prioritization of health promotion for other populations in these countries, lack of resources to implement community health programs, or both. Nevertheless, the representation of countries with younger populations and less developed economies (including Bangladesh, Brazil, China, Indonesia, Mexico, and Taiwan) is an indicator of the seriousness with which population aging is treated globally. Population pyramids in these countries are heavily skewed towards younger age categories, in contrast to countries representing the global North, but also are indicative of a shift towards falling birth rates, predicting accelerated future population aging[54]. Thus, it is consistent with the predictions of the population transition model that interest in programs addressing aging-related concerns is growing in countries where these conditions prevail.

The scope and nature of the community health programs evaluated is extremely variable, ranging from local health screening initiatives to national programs establishing elder-care focused health centers. While some disparities exist between developed and developing, it is noteworthy that large scale and relatively resource intensive programs were evaluated in Taiwan and Indonesia, which are both characterized as developing economies by the United Nations [55]. Some cultural differences are also evident in the design of programs for the care of older adults. For example, Japanese community health responses appear to place emphasis on strategies such as promoting family caregiving [43] and encouraging other community members to act as informal screeners identifying older adults with potential health problems [56], perhaps reflecting the nation’s relatively collectivistic cultural orientation. By contrast, approaches in Western nations such as the United States [24, 25], Canada [57], and Australia [42] appear to be more focused on providing support to allow older adults to continue living alone, perhaps reflective of their relatively individualistic cultural orientation.

Beyond the nature of the health interventions themselves, the other major issue raised in this review is the extent to which systematic program evaluation methodologies have been applied to these programs. It is evident that the number of evaluation studies identified for this review is likely to be small in relation to the number of programs that are likely to exist throughout the world. Because the primary audience for these evaluations is likely to be localized to the places where the corresponding programs are implemented, it is likely that at least some of this disparity can be accounted for by publication of evaluations in languages other than English (which fall outside of the inclusion criteria for this review). However, it is also likely that many programs either do not undergo formal evaluation or are evaluated internally without submission to a peer-reviewed publication. Both of these possibilities raise potential problems, because it makes it difficult to judge the efficacy and cost-effectiveness of these measures.

The evaluation methodologies used were highly varied, and it is important to acknowledge the ways in which the nature of the evaluation may impact judgements about program efficacy. For example, a program may have high participant satisfaction but fail to reduce medical service use. Most of the studies reviewed included evaluation of only a single program outcome or dimension. Because the evaluation methodologies applied were so highly varied, it is important to use caution when attempting to draw comparisons between programs in terms of efficacy. Broadly speaking, it appears that most of the evaluations were generally positive and that programs requiring a greater investment of resources tended to be the most successful. For example, programs bringing multiple types of care and/or other services into a single center [18–21, 26, 37, 50] appeared to be evaluated more positively on the whole than programs that focused on solely on case management to coordinate care between disconnected providers [21, 40, 42, 58–60]. This observation was also supported by one of the few studies to contrast evaluations of alternative programs, finding that participants in the PACE program had better outcomes than participants in another, less intensive, US federal program providing only coordination of care [21]. Narrowly-targeted interventions aimed at specific risks such as fall prevention [48–51] and suicide prevention [53] also appeared to have positive impacts. There was less evidence in support of educational programs in terms of impacting health outcomes.

Funding was a common barrier to program success cited in a wide variety of contexts. Evaluators as far removed as the United States [44], Italy [61], and Indonesia [62] all noted that a failure to fully fund specific programs for older adults prevented them from being fully implemented on the national level, and led to geographical disparities in program access. Others noted that uncertain availability of funding to continue a project beyond its pilot period put program sustainability in jeopardy [51]. Barriers to success noted in other studies included lack of public knowledge about program availability [17] and lack of awareness of aging issues among the older adult population [63].

India’s National Programme for the Health Care of the Elderly (NPHCE) was not evaluated in any of the studies that met the criteria for inclusion in this review. The NPHCE is an ambitious public and community health program, which ultimately envisions the establishment of programs ranging from the local to the regional level, with diverse specific aims ranging from health education and case management to provision of geriatric facilities [15]. The legislative mandate for this program was established in 1999, but has seen only gradual implementation, as a result of budgetary constraints and other barriers [64]. Based on the experiences of programs initiated in other countries reviewed here, this would seem to be a promising set of approaches, although long-term evaluation will be necessary to establish its impact. For example, the NPHCE implementation plan shares similarities with the PACE [18] and HAN [65] programs in the United States, and the IHSP-Elderly program in Indonesia [37], which have been evaluated positively within the literature covered in this review.

These results can be construed as being consistent with the predictions of the population transition model. In particular, whereas the earlier literature is dominated by programs representative of the global North, there is greater evidence of interest in community health programs serving older adult populations in the global South in more recent years. This is likely to be reflective of nascent trends towards population aging in these contexts. As the global population continues to age, it is likely that these trends will continue and will expand further in regions where population aging has not yet begun to be in evidence. Consequently, it is especially important to learn from the programs currently implemented in these regionsin order toinform successful design in the future. Useful insights for gerontological practice may include the observations that the most effective programs appeared to be those that were either narrowly targeted at interventions in specific behavioral domains (e.g., fall prevention) or those that involved resource-intensive coordination of care across medical service domains. These may be areas of particular interest in terms of program development, in comparison with broader health education and case management programs, which tended to have more ambivalent evaluations.

Limitations of this study include the exclusion of sources not published in English and those published in sources other than peer-reviewed journals. This creates potential geographic limitations, as well as excluding evaluations that may have been disseminated internally within an organization, or externally in a report or other format not covered here. Additionally, it is not possible to account for the extent of publication bias. Researchers may be less likely to seek to publish negative or inconclusive evaluation results, particularly when they are involved directly in the design and/or implementation of the program in question. Finally, promising novel approaches such as voluntary banking systems in which younger adults “deposit” volunteer time providing services for older adults that they can “withdraw” were not evaluated in studies that qualified for inclusion in this review, but nevertheless may be valuable to consider in future research.

Nevertheless, this review highlights some important facets of the literature regarding community health programs aimed at older adults and their evaluation. First, programs outside of North America, Europe, and Japan are dramatically under-represented in the evaluation literature; more research outside of these contexts is called for. Second, many published evaluation projects in this area rely on a single methodology or a single outcome; the literature would be strengthened by research employing multiple methods and a broader set of outcomes when conducting this type of evaluation research. As the global population continues to age at a rapid pace, there will be an increasing demand for information about what approaches are most effective for enhancing care of older adults as well as for reducing the economic burden of providing that care. The literature reviewed in this paper provides substantive evidence in favor of some specific approaches, although much work remains to delineate the most effective approaches, particularly in under-represented global populations.

## Conclusion

Community health programs have the potential to address some of the challenges associated with population aging, as supported by the findings of evaluations conducted in many different national contexts and summarized in this review. It is important to include program evaluation as a component of future community and public health interventions aimed at aging populations to better understand how to improve these programs and thereby help meet these needs as they evolve throughout the globe.
